# Falmouth Sanitation

**Published:** 1900-03-03

**Authors:** 


### The Hospital

A Journal of

The Medical Sciences and Hospital Administration.

Vol. XXVII.—No. 701.] Edited by Sir Henry Burdett, K.C.B., and Solomon C. Smith, M.D., M.R.C.P. [March 3, 1900.

### FALMOUTH SANITATION.

The local authorities of Falmouth, by the pen of their representative in the House of Commons, Mr. F. J. Horniman, M.P., have been in communication with the Admiralty concerning the removal of the training ship "Ganges" from Falmouth to Harwich, and Mr. Horniman has declared that, owing to the removal of the "Ganges" from Falmouth Harbour, a considerable amount of distress has been caused in the town, the absence of the ship being a direct loss to a number of small traders, besides being felt in other directions. Mr. Horniman asked if it were not possible for a second-class cruiser to be stationed in the harbour for the purpose of training the Naval Reserve. The Lords Commissioners, in their reply, are content to state that her Majesty's ships are of necessity primarily stationed according to the requirements of the naval service, apart from the question of the advantage accruing to local trade from their presence at any particular port, and to express regret that the exigencies of the service will not admit of compliance with Mr. Horniman's request. All this, of course, is within the limits of the strictest official propriety, and has the advantage, from the official point of view, of admitting of no further reply or argument. We cannot, however, entirely divest ourselves of a suspicion, or almost of a hope, that the polite "non possumus" of their Lordships may to some extent be due to another official document which has appeared almost at the same time as the correspondence, in the shape of a report by Dr. G. S. Buchanan on a recent prevalence of enteric fever at Falmouth, just issued by the Medical Department of the Local Government Board. It appears from that report that dropping cases of fever appeared in Falmouth during every month of the first half of last year, and that these were preliminary to a very considerable outbreak in August and September, the causes of which Dr. Buchanan was directed to investigate on behalf of the Department. His report indicates a condition of things which might have been the result of diabolical ingenuity, carefully exerted for the express purpose of promoting the diffusion of disease. Nothing seems to have been right. There were some waterworks deriving at least a part of their supplies from gathering grounds saturated with pollution. The construction of the house drains, and their connections with the sewers, were perhaps as bad as it was possible for ignorance and slovenliness to make them; and a certain expanse of foreshore in the harbour appears to have become a sort of drying ground for an overflow of offensive sludge from a sewage tank. An attempt was made some time ago to arrange for the purchase of the waterworks by the municipality; but this attempt was defeated, probably, as Dr. Buchanan suggests, because the ratepayers had no confidence in the authorities upon whom, in that case, the management of the undertaking would have devolved. A brand-new Town Council is said to have come into existence in 1892, but it has so far done nothing, and appears to be much the same thing as its predecessor, only "more so." Dr. Buchanan points out that the future prosperity of Falmouth, whether as a health resort, a watering place, or a place of business, seems to be contingent upon its obtaining a Town Council prepared to grapple boldly with the conditions which he describes, and to spend money, under expert advice, for the purpose of removing the perils by which its inhabitants are now beset. The residents have received a warning on which it will be wise for them to act, and to act while there is yet time to prevent the occurrence of still greater evils.

Falmouth, properly drained and with a protected water supply, is a place which should present many attractions alike to invalids and to holiday-makers. Its mild and equable climate, its noble harbour, and the beauty of the surrounding scenery are all advantages which would be calculated to give it a high position among seaside places of resort, if they were not more than neutralised by the stupidity and mismanagement of its local authorities. Whether the action of the Admiralty, in removing the training ship, was really consequent upon Dr. Buchanan's report, is a matter as to which no certainty can be attained by anyone beyond the sacred precincts of Whitehall; but it is at least possible that this may be the correct interpretation, and it is unquestionably to be hoped that it will be so regarded in the town itself. If such consideration for the health and safety of our future sailors has indeed been shown, it would be a ground for additional confidence in the general management of the Admiralty, and would encourage us to hope that, whatever the future may have in store for us, the efficiency of our first line of defence will be as complete in all other respects as in matters of sanitation. In the meanwhile, the people of Falmouth should make haste to set their houses in order; and, when this has been done, they might perhaps make application for a cruiser with more reasonable prospects of success.

